# The Id-protein family in developmental and cancer-associated pathways

**DOI:** 10.1186/s12964-016-0161-y

**Published:** 2017-01-25

**Authors:** Cornelia Roschger, Chiara Cabrele

**Affiliations:** Department of Molecular Biology, University of Salzburg, Billrothstrasse 11, Salzburg, 5020 Austria

**Keywords:** Id protein, Helix-loop-helix protein, Intrinsically disordered protein, Cell-cycle regulation, Cell differentiation, Development, Chemoresistance, Cancer stemness

## Abstract

Inhibitors of DNA binding and cell differentiation (Id) proteins are members of the large family of the helix-loop-helix (HLH) transcription factors, but they lack any DNA-binding motif. During development, the Id proteins play a key role in the regulation of cell-cycle progression and cell differentiation by modulating different cell-cycle regulators both by direct and indirect mechanisms. Several Id-protein interacting partners have been identified thus far, which belong to structurally and functionally unrelated families, including, among others, the class I and II bHLH transcription factors, the retinoblastoma protein and related pocket proteins, the paired-box transcription factors, and the S5a subunit of the 26 S proteasome. Although the HLH domain of the Id proteins is involved in most of their protein-protein interaction events, additional motifs located in their N-terminal and C-terminal regions are required for the recognition of diverse protein partners. The ability of the Id proteins to interact with structurally different proteins is likely to arise from their conformational flexibility: indeed, these proteins contain intrinsically disordered regions that, in the case of the HLH region, undergo folding upon self- or heteroassociation. Besides their crucial role for cell-fate determination and cell-cycle progression during development, other important cellular events have been related to the Id-protein expression in a number of pathologies. Dysregulated Id-protein expression has been associated with tumor growth, vascularization, invasiveness, metastasis, chemoresistance and stemness, as well as with various developmental defects and diseases. Herein we provide an overview on the structural properties, mode of action, biological function and therapeutic potential of these regulatory proteins.

## Background

The helix-loop-helix (HLH) transcription factors are a large family of proteins that share a common HLH domain for protein-protein interaction. They may be further divided in seven classes (Table 1) [1–3]: class I proteins are broadly expressed and contain an additional basic DNA-binding motif N-terminal to the HLH domain, thus they are called basic-HLH (bHLH) proteins. Examples of class I bHLH transcription factors are the E proteins (E12, E47, HEB and E2-2, also known as transcription factor 4, abbreviated as TCF-4, or immunoglobulin transcription factor 2, abbreviated as ITF-2 [4]). The E proteins can form DNA-binding homodimers or heterodimers with other E proteins and class II bHLH proteins like the tissue-specific myogenic regulatory factors (e.g. MyoD) [5, 6]. In class III and IV proteins the bHLH domain is C-terminally elongated, respectively, with a leucine-zipper (LZ) motif [2, 7–11] and a Per-ARNT-SIM (PAS) motif [12]. Class VI proteins contain proline residues in the DNA-binding motif [2, 13, 14]. Finally, class V HLH proteins are the inhibitors of DNA binding and cell differentiation (Id1-4) that display no DNA-binding motif [15–17].

**Table 1:** Class I-VII of the HLH transcription factors

Class	Structural domain	Representative members	Properties and function
I	bHLH	E-protein family (E12, E47, E2-2/TCF-4/ITF-2, HEB) [1–3, 333]	Broadly expressed; Self- or heteroassociation with class II proteins; Regulation of neuro-, myo-, lymphogenesis
II	bHLH	Myogenic regulatory factors (MyoD, myogenin, Mrf4, Myf-5,6) [6]; NeuroD/Beta2 [334]; Mash-1 [335, 336], d/e-HAND [337]; Twist [338]	Tissue-specific; Heteroassociation with class I proteins; Regulation of myogenesis (myogenic regulatory factors), islet cells differentiation (NeuroD/Beta2), neurogenesis (Mash-1), cardiac morphogenesis (d/e-HAND), and mesoderm development (Twist)
III	bHLH-LZ	MiT family (MiTF, TFE3, TFEB, TFEC) [9, 10]; Myc [8]	Melanocytes maturation (MiTF), B cells activation (TFE3), placental vascularization (TFEB), osteoclast development (MiTF, TFE3, TFEC), cell proliferation/differentiation, oncogenesis and apoptosis (Myc)
IV	bHLH-LZ	Mad family (Mad1/3/4, Mxi1) [339–341], Max [7, 342]	Homodimerization or heterodimerization with Myc; Regulation of cell proliferation
V	HLH	Id proteins (Id1-4) [16, 17, 24, 343]	Heterodimerization with class I and, to a minor extent, class II proteins; Regulation of cell proliferation/differentiation during development and in cancer
VI	b(Pro)HLH	HES family (HES1-7) [13]	Regulation of cell proliferation/differentiation during embryogenesis
VII	bHLH-PAS	AhR, HIFα, SIM, ARNT [12]	Regulation of xenobiotic (AhR/ARNT) and hypoxic (HIFα/ARNT) response genes, and of neural development (SIM/ARNT)

**Fig. 1:**
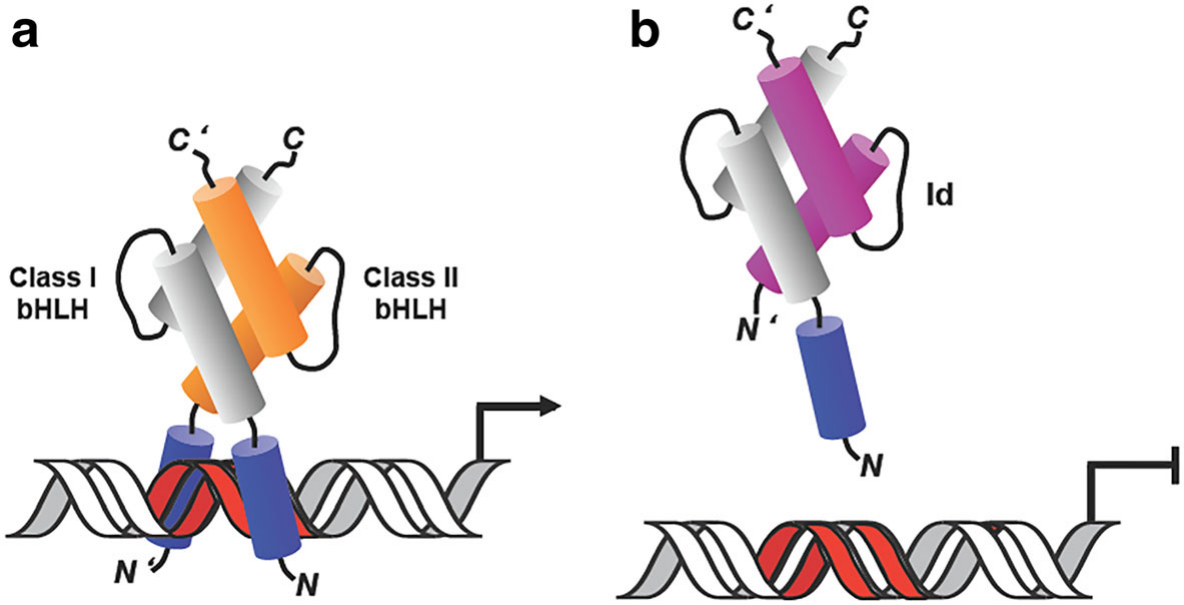
Control of DNA transcription by positive and negative HLH regulators. **a** Broadly expressed bHLH E proteins build heterodimers with tissue-specific bHLH proteins, which results in DNA binding at specific DNA sequences (in *red*) and DNA-transcription activation. **b** E-protein association with the Id proteins results in dimers with no ability to bind the DNA because the Id-protein subunit lacks the DNA binding region, which leads to inhibition of DNA-transcription activation. The helices of the HLH domains are represented by cylinders (*grey* for class I bHLH, *orange* for class II bHLH and *magenta* for Id); a blue-colored cylinder represents the basic DNA-binding region of the bHLH domain

The HLH domain, which consists of two amphipatic α-helices connected by a loop, is responsible of the homo- or heterodimerization of the (b)HLH proteins: the resulting fold is a non-covalent, parallel, left handed four-helix bundle [5, 18]. In the case of bHLH dimers, such fold allows the tweezers-like juxtaposition of the two N-terminal basic helices, which is ideal to specifically bind DNA double strands including E-boxes (CA*NN*TG) [19–21], N-boxes (CAC*N*AG) [14], and Ets sites (GGAA/T) [22] (Fig. 1). Formation of the ternary complex DNA[bHLH(−LZ)]_2_ triggers transcriptional activation. This, however, does not occur for class V proteins, as they lack the DNA-binding motif: as a result, these proteins may sequester class I and II bHLH proteins in non-DNA-binding dimers, thus acting as negative regulators of bHLH-mediated gene expression [15, 23].

This review will focus on the class V Id proteins, with the aim to give an overview of them, discussing the following aspects: (i) structural features, (ii) mode of action, (iii) biological function in physiological as well as pathological scenarios, and (iv) potential role in tumor therapy.

## Structural features

**Fig. 2:**
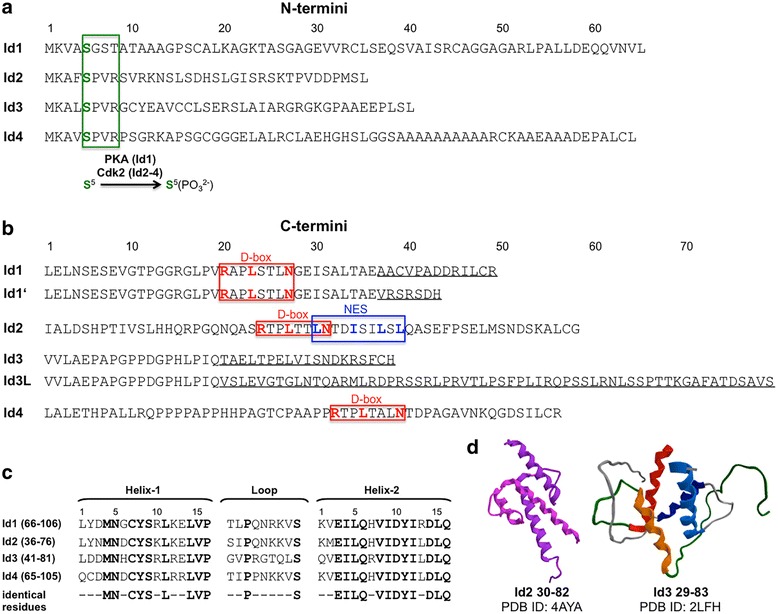
Amino-acid sequences of the N-terminal (**a**) and C-terminal domains (**b**) as well as of the HLH domains (**c**) of the human Id proteins (for Id1 and Id3 the C-terminus found in a spliced form is reported as Id1′ and Id3L). **d** Structures of the homodimers of the fragments Id2 30–82 [55] and Id3 29–83 [56]. D-box, destruction box; NES, nuclear export signal (UniProtKB: P41134-1 for Id1, P41134-2 for Id1′, Q02363 for Id2, Q02535 for Id3, P47928 for Id4. GenPept: S71405 GI: 2135331 for Id3L)

The Id1 protein was first identified in 1990 by Benezra et al. [15]. Since then four mammalian Id proteins, Id1-4 [24–26], as well as *Drosophila* [27] and *Xenopus* [28] homolog proteins have been identified. In humans the four Id genes are located on chromosomes 20q11 (Id1) [29, 30], 2p25 (Id2) [29], 1p36.1 (Id3) [31, 32], and 6p21-p22 (Id4) [33]. For mouse, rat and human Id1 [30, 34–38] as well as for rat and human Id3 [39, 40] a spliced form has been also detected, which differs from the canonical one only in the C-terminal domain (Fig. 2b): for example, the canonical and spliced forms of human Id1 are 155- and 149-residue long and differ from position 143 [30, 36, 38]. The canonical and spliced forms of human Id3 are 119- and 160-residue long and differ from position 101 [39]. Interestingly, the spliced form of Id1 has much higher propensity to homodimerize than the canonical form [37]. Instead, the spliced form of Id3 seems to have less affinity for the bHLH E protein E47 than the canonical form [39].

Sequence alignment of the four Id proteins reveals that the HLH domain is highly conserved, especially within the two helical motifs (helix-1 and helix-2) and at their junctions with the loop (Fig. 2c). Accordingly, the Id HLH region badly tolerates sequence modifications, resulting in altered conformation [41–44] and function [45]. Contrarily to the highly conserved HLH domain, the N-terminal and C-terminal domains are unique for each of the Id proteins, being different both in length and amino-acid sequence (Fig. 2a, b). Nevertheless, some common features can be found also in these regions: for example, Id1-4 possess a phosphorylation site at Ser-5 [46–49], and Id1,2,4 display a C-terminal destruction box (D-box) that triggers protein degradation via the anaphase-promoting complex/cyclosome Apc/C and its activator Cdh1 (Apc/C^Cdh1^) [50]. Instead, only Id2 contains a nuclear export signal (NES) that is recognized by the nuclear export receptor CRMP1 [51].

The HLH and flanking regions display different structural properties: indeed, the Id HLH domain undergoes self- (Id2 [52], Id3 [53, 54]) or heteroassociation with the HLH domains of class I and II proteins and folds into a four-helix bundle, as shown by the crystal structure of the Id2 HLH domain (PDB ID: 4AYA) [55] and by the solution NMR structure of the Id3 HLH domain (PDB ID: 2LFH) [56] (Fig. 2d). In contrast, the N-terminal and C-terminal domains are mainly disordered, as suggested by disorder probability prediction analysis [57–59] as well as by conformational data on synthetic peptides reproducing the N-terminal and C-terminal regions of the Id proteins [42, 43]. Therefore, the Id proteins contain intrinsically disordered regions that, in the case of the HLH region, undergo folding upon self- or heteroassociation [41, 44, 53, 55, 56]. A common feature of intrinsically disordered proteins is the high propensity to aggregate and precipitate, which represents the major obstacle for the structural investigation of these proteins. Indeed, attempts to grow crystals of the full-length Id2 protein failed thus far [60], and only N-terminal and C-terminally truncated Id2 (residues 30–82) was successfully crystallized [55].

Despite the lack of a well-defined structure, the N-terminal and C-terminal domains are certainly important for the biology of the Id proteins, as supported by the presence of regulatory signals there, including ubiquitination, phosphorylation and molecular recognition elements like NES and D-box, which will be discussed in the following. In addition to the primary structure and its post-translational modifications, it is highly probable that also the conformational dynamics of the flexible regions will play a role in protein-protein interaction events.

## Mode of action

The Id proteins perform their biological function via protein-protein interactions that involve not only class I and II bHLH proteins [61–63], but also proteins belonging to other families and containing other types of domains. These include the retinoblastoma protein tumor suppressor (pRb) and related pocket proteins [64, 65], the actin-associated protein enigma homolog (ENH) [66, 67], the p200 family member p204 [68, 69], Ets-domain proteins [70], MIDA1 [71, 72], Pax transcription factors [73], adenovirus E1A proteins [74], ADD1/SREBP-1c [75], the C8 subunit of the 20 S proteasome [76], the hepatitis B virus-encoded protein X (HBX) [76], the S5a subunit of the 26 S proteasome [77], the COP9 signalosome (CSN) subunits CSN5 and CSN7 [78], the deubiquitinase USP1 [79], the Apc/C subunits Apc1, Apc5, Apc8/Cdc23 [50], the cell-membrane protein caveolin-1 [80], the four-and-a-half LIM-only protein 2 (FHL2) [81], the Von-Hippel Lindau(VHL)-elongin-C complex [82], and the estrogen receptor beta-1 (ERβ1) [83] (Table 2). The fact that some of these interactions are specific for individual Id-family members suggests that they are not only mediated by the highly conserved HLH motif, but also by the less conserved N-terminal and C-terminal regions. For example, the interaction of Id2 with the VHL-elongin-C complex is suggested to occur in a short region of the Id2 N-terminal domain, which contains Thr-27: however, Dyrk1-mediated phosphorylation of Thr-27 prevents the interaction with the protein complex [82].

**Table 2:** Protein-protein interactions involving Id proteins

Id protein	Protein partner	Description	References
Id1-4	Class I and II bHLH proteins	Dominant negative regulation of bHLH factors	[61–63, 236]
Id2	pRb and related pocket proteins p107 and p130	Inhibition of pRb-mediated cell-cycle arrest (as no interaction between the pRb small pocket and Id2 was detected by mass spectrometry and NMR spectroscopy [136], a multiprotein complex is likely to be formed)	[64, 65]
Id2	ENH	Id-protein localization in the cytoplasm by association of the Id2 HLH and ENH LIM domains	[66, 67]
Id1-3	p204	Id-protein localization in the cytoplasm	[68, 69]
Id1-3	Ets-domain proteins Elk-1 and SAP-1/-2	Inhibition of the winged-helix-turn-helix transcription factors regulating the expression of immediate-early response genes such as *c-Fos* and *Egr*1	[70]
Id1	MIDA1	Inhibition of MIDA1-Z-DNA interaction with stimulation of cell growth and inhibition of neural differentiation	[71, 72, 344, 345]
Id1-3	Pax-2/-5/-8	Inhibition of the paired-box transcription factors involved in development	[73]
Id1, Id2	Adenovirus E1A protein	Induction of apoptosis in cells expressing p53 mutants	[74]
Id2, Id3	ADD1/SREBP-1c	Inhibition of the bHLH-LZ transcription factor that regulates the expression of adipocyte genes	[75]
Id1	C8, HBX	Induction of proteasome-mediated HBX degradation	[76]
Id1	S5a	Suppression of the Id-protein activity	[77]
Id1, Id3	CSN5 (Id1, Id3), CSN7 (Id3)	Suppression of Id-protein ubiquitination by CSN-mediated phosphorylation	[78]
Id1-3	USP1	Id-protein deubiquitination	[79]
Id2	Apc/C subunits Apc1, Apc5, Apc8/Cdc23	Id2-protein degradation	[50]
Id1	Caveolin-1	Induction of cell migration and chemotherapy resistance in prostate cancer	[80]
Id1-4	FHL2	Antagonism of the inhibitory effect of the Id proteins on E47-mediated transcription	[81]
Id2	VHL-elongin-C	Inhibition of ubiquitination and degradation of HIF2α	[82]
Id1	ERβ1	Inhibition of cell proliferation and *p21* up-regulation	[83]

Interestingly, the presence of the unique polyalanine segment within the N-terminus of Id4 seems to positively affect the HLH-mediated interaction of the Id4 protein with the other Id proteins [63], an observation that further underlines the structural and functional importance of the N-terminal and C-terminal domains of the Id proteins.

## Regulation

For the correct function of a protein in the cell, its expression, localization, and degradation must be strictly timely regulated. The following subsections report on the known mechanisms that regulate the Id-protein activity.

## Gene expression

**Fig. 3:**
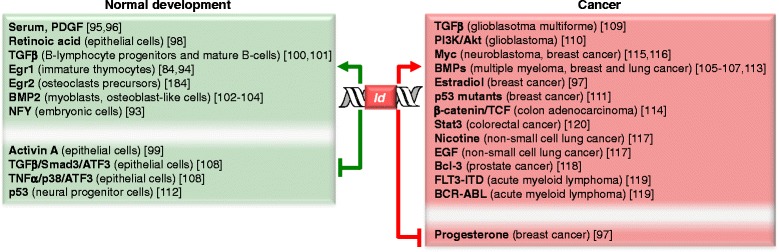
Selected molecules/pathways involved in the regulation of *Id* gene expression in normal development and cancer

Usually, *Id* gene expression is positively regulated in undifferentiated, highly proliferative, embryonic or cancer cells [16, 84–91] (Fig. 3). For example, during development the *Id* gene expression is activated in stem and progenitor cells to support proliferation and inhibit differentiation, whereas it is repressed upon lineage commitment and differentiation [1, 92]. Recently, it has been shown that the *Id1-3* genes are targets of the nuclear factor Y (NFY) complex (NFYa-c) that binds the CCAAT box on their promoters (in contrast, the *Id4* gene lacks the CCAAT box) [93]: accordingly, NFY incorporation into the *Id1-3* gene promoters decreases upon induction of differentiation with retinoic acid of the human embryonic carcinoma cell line NTera2, which is a valuable cell model to study the expression profile during development. This is due to the loss of NFYc at the protein level in the differentiating cells, which results in *Id1-3* genes down-regulation. Interestingly, also the epigenetic markers for gene transcription (histone 3 Lys-9 acetyl, H3K9ac) and repression (histone 3 Lys-9 dimethyl, H3K9me2) incorporated into the *Id1-3* gene promoters respectively decrease and increase during differentiation. However, after 7 days of differentiation the positive regulators NFYc and H3K9ac recover with consequent increase in *Id1-3* gene expression that is required for cellular growth [93].

In primary immature thymocytes the *Id3* gene expression is activated by the Egr1 transcription factor that is a downstream target of the TCR-mediated activation of the RAS-ERK-MAPK pathway, which promotes thymocyte maturation upon inhibition of the E proteins [84, 94].

In vitro, treatment of cells with serum or platelet-derived growth factor (PDGF) induces *Id* gene expression [95] (serum-induced expression of *Id1* is linked to the serum-dependent protein complex including the Egr1 protein [96]). Also the sex hormone estradiol positively affects *Id1* gene expression in human breast cancer cells, which, however, may be counteracted by progesterone [97]. *Id1* and *Id3* gene expression can be stimulated by retinoic acid in keratinocytes [98]. In contrast, the cytokines activin-A and TGFβ1 lead to suppression of *Id1*, *Id2* and *Id3* gene expression in keratinocytes [99]. However, TGFβ1 has been shown to induce *Id3* gene expression in B-lymphocyte progenitors, resulting in inhibition of their growth and survival [100], as well as *Id2* gene expression in mature B cells, preventing IgE class switching [101]. Bone morphogenic protein 2 (BMP2), another cytokine from the TGFβ family, positively affects *Id1* gene expression in myoblasts [102, 103], osteoblast-like cells [104], breast [105] and lung [106, 107] cancer cells. In epithelial cell lines, the BMP2-mediated *Id1* gene expression may be counteracted by the TGFβ/Smad3 or TNFα/p38 pathways that activate the stress response factor and transcriptional repressor ATF3 [108]. Instead, TGFβ does not induce ATF3 in glioblastoma multiforme, which turns the TGFβ-mediated transcription of *Id1* from repression to activation [109]. In glioblastoma cells Id1 expression is also PI3K-dependent through the phosphorylation of 4E-BP1 via Akt-mTORC1 or Akt-PPM1G. Increase in the phosphorylation state of 4E-BP1 results in the activation of Id1 translation, leading to increased Id1 expression and glioblastoma malignancy [110].

Mutations of p53 have been shown to positively regulate the transcription of the *Id4* gene in breast cancers: indeed, complexes of p53 mutants and E2F1 bind to the *Id4* promoter and activate *Id4* expression [111]. Instead, *Id2* expression is down-regulated by the transcriprional repressor p53 in neural progenitor cells [112].

In multiple myeloma the *Id1* and *Id2* gene expression is induced by over-expressed BMPs, which supports cell proliferation [113]. In colon adenocarcinoma the *Id2* gene is a target of the β-catenin/T-cell factor transcription pathway and induces the clonogenic growth of the colon cells [114]. In neuroblastoma the *Id2* gene is activated by Myc oncoproteins, which leads to the inhibition of the pRb tumor suppressor pathway with consequent cell-cycle progression [115]. In breast cancer Myc up-regulates Id3 that supports the entry in the S-phase by enhancing the cyclin/Cdk activity [116]. In non-small cell lung cancer the activation of the *Id1* gene promoter can be induced by nicotine and EGF in a Src-dependent manner, which leads to the down-regulation of ZBP-89, a zinc finger transcriptional repressor of the mesenchymal markers fibronectin and vimentin [117]. In prostate cancer the over-expression of B-cell leukemia 3 (Bcl3) protein is correlated with the expression of Id1 and Id2, which is in turn accompanied with resistance to pro-apoptotic drugs [118]. In acute myeloid leukemia Id1 has been shown to be a target of the oncogenic tyrosine kinases FLT3-ITD and BCR-ABL, which results in protection of the cells against TRAIL-induced apoptosis [119]. In colorectal cancer Stat3 mediates the transcriptional activation of the *Id1* gene, which correlates with p53 inactivation [120].

## Phosphorylation

**Fig. 4:**
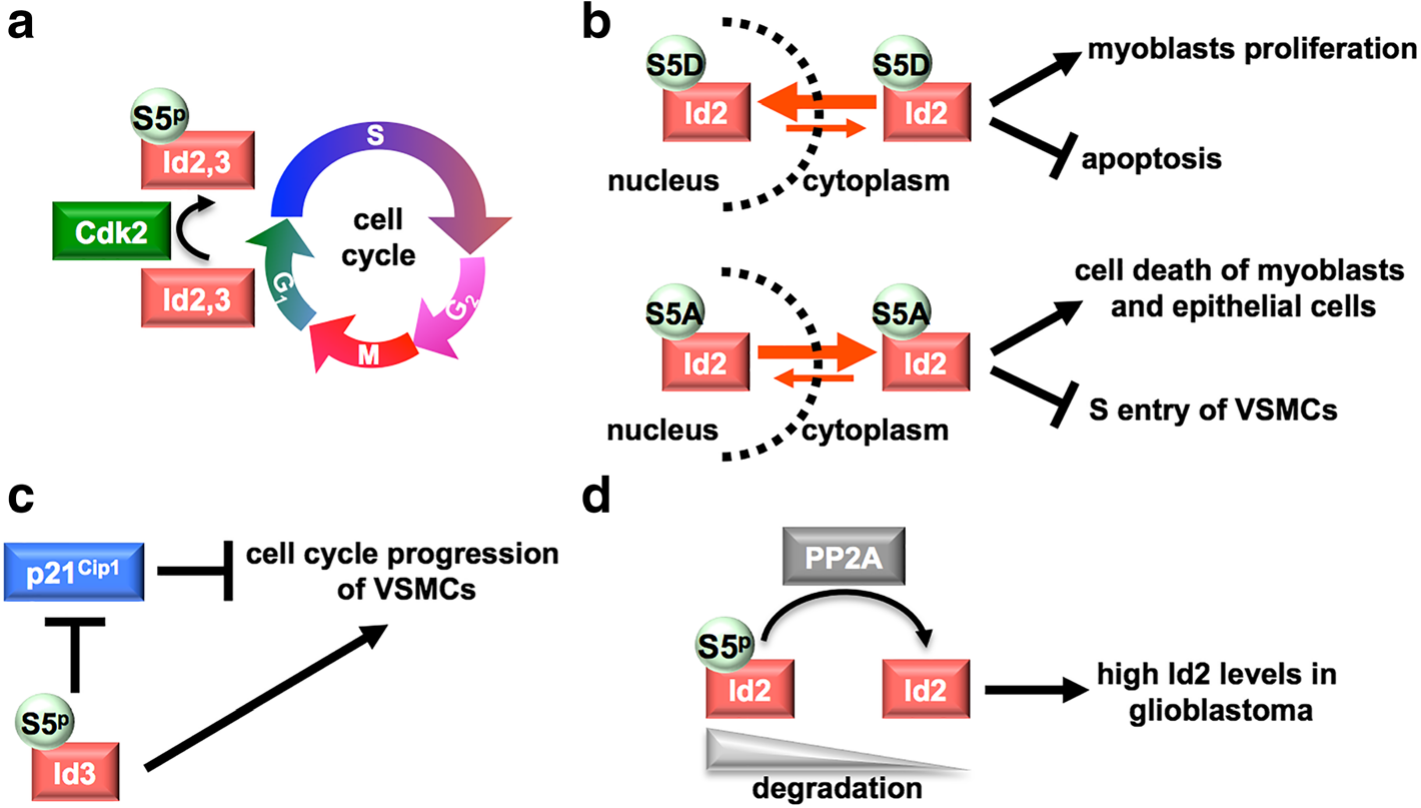
Phosphorylation of Id2 and Id3. These proteins are phosphorylated by Cdk2 at the G_1_-S transition [46–48, 121–123] (**a**). Phosphorylated Id2 (or its S5D mimic) accumulates in the nucleus, whereas phosphoablated Id2 (S5A) accumulates in the cytoplasm and induces cell-cycle arrest or apoptosis [121, 122] (**b**). Phosphorylated Id3 induces cell-cycle progression of VSMCs via inhibiting the cell-cycle blocker p21^Cip1^ at the transcriptional level [123] (**c**). Phosphorylated Id2 is less resistant to degradation than unphosphorylated Id2. In glioblastoma high Id2 levels can be maintained by PP2A activity (**d**) [124]

Id2 and Id3 may be phosphorylated at Ser-5 by Cdk2 at the G_1_-S transition [46–48, 121–123] (Fig. 4). Phosphorylated Id2 accumulates in the nucleus and seems to be key player in cell-cycle regulation, as the phosphoablated mutant (Ser-5-Ala-Id2) induces apoptosis of myoblasts [121] and mammary epithelial cells [122], as well as it inhibits the entry in the S-phase of vascular smooth-muscle cells (VSMCs) [123]. Also Id3 phosphorylation is important for VSMCs proliferation: it has been proposed that phosphorylation of Id3 at the G_1_-S transition contributes to suppress the Cdk2- and cell-cycle-blocker p21^Cip1^ in the early G_1_ phase, further supporting the linkage between Id3 phosphorylation and cell-cycle progression [47]. The negative effect of Id3 phosphorylation on the p21^Cip1^ levels is probably due to enhanced ability of the phosphoprotein to inhibit transcriptional activation of *p21* ^*Cip1*^ in comparison to the phosphoablated (Ser-5-Ala-Id3) and non-phosphorylated protein, an assumption that may be justified by the observation that Ser-5 phosphorylation seems to affect the protein-protein interaction properties of Id3 (as well as of Id2), at least with respect to the class I bHLH proteins [46–48].

In neural precursor cells the N-terminal Id2 phosphorylation regulates the expression level of the protein: indeed, a phosphoablated mutant appears to be protected against proteasome-dependent degradation, thus promoting proliferation. Moreover, the phosphatase PP2A has been shown to maintain high Id2 levels in glioblastoma: in contrast, inhibition of selected PP2A subunits in glioblastoma-derived stem cells (GSCs) decreases Id2 levels as the result of regained protein phosphorylation and enhanced degradation [124].

Unlike Id2 and Id3, Id1 has been found to be phosphorylated at Ser-5 by PKA, which apparently blocks the nuclear export of the protein [49].

## Degradation

The Id proteins are short-living proteins with half-lives shorter than one hour [125, 126]. The proteins Id1-3 are degraded via the 26 S proteasome pathway upon N-terminal ubiquitination, whereas Id4 degradation is dependent from the E1 enzyme [125]. Proteasome-mediated degradation of Id1 and Id3 is negatively regulated by the COP9 signalosome (CSN) that directly interacts with the proteins and induces their phosphorylation [78]. Moreover, the deubiquitinase USP1 can associate with and deubiquitinate Id1-3 in mesenchymal stem cells, thus preserving their stem cell state [79]. Also the interaction of the Id proteins with their bHLH binding partners protects them from fast degradation [125–127]. In contrast, degradation of Id1, Id2 and Id4 is triggered by Apc/C^Cdh1^ that recognizes the destruction box (D-box) motif, RXXLXXXN, located C-terminally of the HLH domain (Table 3) [50].

**Table 3:** D-box and NLS/NES motifs of the Id proteins

Id motif	Amino-acid sequence
D-box	
Id1 (126–133)	**R**AP**L**STL**N**
Id2 (100–107)	**R**TP**L**TTL**N**
Id4 (137–144)	**R**TP**L**TAL**N**
NES-like	
Id1 (98–109)	VIDY**I**RD**L**Q**L**EL
Id2 (106–115)	**L**NTD**I**SI**L**S**L**
NLS-like	
Id1 (74–91)	S**R**L**K**ELVPTLPQN**RK**VS**K**
Id2 (44–61)	S**K**L**K**ELVPSIPQN**KK**VS**K**

The D-box motifs are recognized by Apc/C^Cdh1^ and trigger Id-protein degradation. The characteristic D-box amino-acid positions are in bold [50]. The NLS/NES motifs regulate the Id-protein nucleo-cytoplasmic shuttling [51, 126, 129]. The hydrophobic residues important for the nuclear export are in bold. The conserved basic positions in the NLS motifs are in bold

## Subcellular localization

The Id proteins may be found both in the nucleus and in the cytoplasm [128], and their nucleo-cytoplasmic distribution is regulated either by passive diffusion, due to their small size (13–18 kDa), or by nuclear localization/export signals (NLS/NES) embedded in their sequences, which regulate the nucleo-cytoplasmic shuttling by binding nuclear pore complexes (Table 3). Id1 [129] and Id2 [51] contain a NES motif in the HLH domain and in the C-terminus, respectively. Although the Id proteins lack canonical NLS motifs, it is likely that the basic residues present in the HLH domain fulfill the task of nuclear localization, as it has been shown for Id1 [126]. However, in the case of Id3, which lacks the basic-residue pattern shown by Id1 and Id2, the HLH domain seems to be enough for nuclear localization: indeed, Id3 has been shown to accumulate in the nucleus or in the cytoplasm upon co-expression with or in the absence of bHLH E47, respectively [127]. This suggests that the bHLH protein, which contains its own NLS, my act as a carrier of the Id protein into the nucleus. The localization of Id2 in the cytoplasm may be positively regulated by the cytoplasmic protein ENH that is able to interact with the Id2 protein and to retain it in the cytoplasm: the Id2 HLH domain and at least one of the three LIM domains of ENH mediate the interaction between the two proteins [66].

## Cell-cycle regulation

**Fig. 5:**
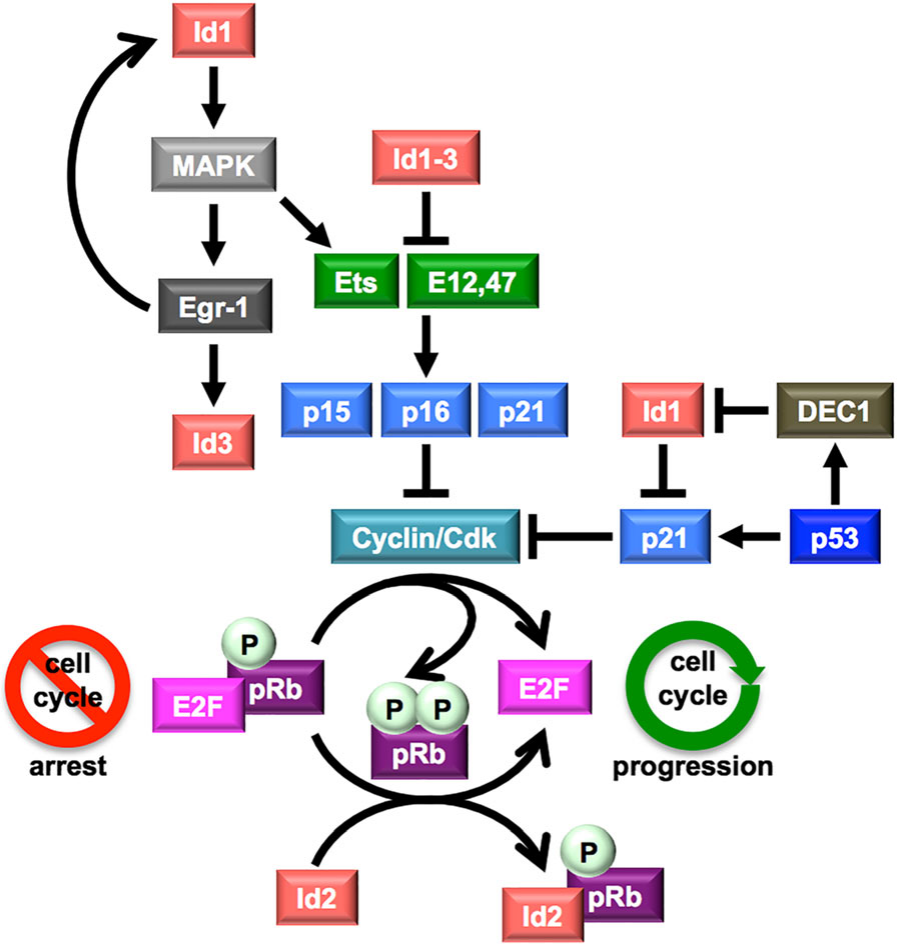
The Id proteins promote cell-cycle progression. Cyclin/Cdk-mediated cell-cycle progression is supported by the Id-protein-induced inactivation of the Cdk inhibitors p15/16/21 and Ets, or by the direct interaction of Id2 with hypophosphorylated pRb. In addition, a cross talk between Id1 and the p53 pathway involving the cell-cycle blocker p21 and the *Id1* transcriptional repressor DEC1 has been proposed. Also, Id1 may activate the MAPK pathway by promoting the phosphorylation of Raf and MEK1/2, which results in the induction of the transcription factor Egr1, a positive regulator of *Id1* and *Id3* gene expression

The Id proteins contribute to the regulation of the cell cycle in the G_1_ phase, mainly by antagonizing the transcriptional activation of differentiation-associated genes like the Cdk inhibitors *p15*, *p16* and *p21* mediated by the class I bHLH proteins [130] and Ets-domain proteins [131], promoting cell-cycle progression [132–134] or inhibiting cell senescence [135] (Fig. 5).

Besides Cdk inhibitors, the Id proteins affect also other cell-cycle regulators, including pRb and p53. Id2, but not the other Id proteins, has been shown to interact with the hypophosphorylated, active form of pRb and with the pRb-related pocket proteins p107 and p130, which abrogates their cell-cyle arrest activity [64, 65]. The mode of interaction between pRb and Id2 is not fully understood and, probably, it includes a multidomain interaction, since the small pocket domain of pRb is not able to bind Id2 in vitro [136]. Unlike Id2 that affects the pRb pathway by physically interacting with the members of the pRb family, Id1 and Id3 are likely to indirectly regulate the pRb activity by binding to Ets-domain proteins [70] or class I bHLH proteins [130] and thus decreasing the expression of p16 [131, 135, 137] or p21 [138], potent inhibitors of the cyclin/Cdk complexes that mediate pRb phosphorylation. Hyperphosphorylated pRb is no longer able to bind the transcription factors E2F1-3 that, thus, activate cell division and may trigger tumorigenesis [139].

Id1 is able to impair the p53-mediated response to DNA damage, which has been attributed to the negative effect of Id1 on p21 expression; in contrary, p53 up-regulates the bHLH transcription factor DEC1 that, in turn, down-regulates Id1 [138]. This indicates a cross talk between p53 and Id1, which regulates cell-cycle arrest and senescence versus cell-cycle progression.

Id-protein induced cell proliferation has been also correlated to activation of the MAPK (mitogen-activated protein kinase) signaling pathway [140]. Indeed, Id1 promotes not only phosphorylation of Raf and MEK1/2, which are key regulators of the MAPK pathway, but also Egr1 expression, which is a downstream effector of the activated MAPK pathway and, in turn, activates the transcription of the *Id1* gene [96]. This suggests that Id1 is not only a positive upstream regulator but also a downstream target of the MAPK signaling pathway [96, 140]. Also the *Id3* gene is a downstream target of Egr1 upon TCR activation of the ERK MAPK pathway [94].

## Id proteins in developmental processes and disorders

The Id proteins play a key role during development [16, 86, 91, 141]. Based on *Id* gene knockout animal models, single knockout leads to developmental defects of different entity, depending on the lacking *Id* gene [142–148] (Table 4). Instead, the double knockout of *Id1* and *Id3* leads to embryonic lethality [149]. This suggests that Id3 can compensate loss of Id1 during embryonic development.

**Table 4:** *Id* gene knockout studies in mice

Deleted gene	Phenotype
*Id1−/−*	Decreased neovascularization [146]
*Id2−/−*	Lack of Langerhans and splenic dendritic cells, reduced number of NK cells, altered peripheral lymphoid organs [143, 147]
*Id3−/−*	Defects in B-cell activation and proliferation upon B-cell receptor signaling, and in both negative and positive selection of T-lineage progenitors [144, 145]
*Id4−/−*	Altered brain size, defects in forebrain development [142, 148]
*Id1−/−*, *Id3−/−*	Embryonic lethality with cranial hemorrhage, small brain size, premature neural differentiation [149]

## Neural processes

Id1 and Id3 are highly expressed in the early stages of the central nervous system, whereas their levels decrease in the late stages [150–152], which suggests a role of these proteins in stimulating neural precursor cells proliferation while inhibiting differentiation. In the case of Id2 and Id4, their expression is retained also in adulthood [88, 153, 154].

During neural development Id2, E47 and pRb contribute to the regulation of the cell cycle mainly by controlling the Cdk inhibitor p57^Kip2^, which is essential for cell-cycle arrest and differentiation. The transcription of *p57* ^*Kip2*^ is activated by E47 and indirectly repressed by Id2, which inhibits E47. However, pRb may counteract the inhibitory activity of Id2. Therefore, it has been suggested that p57^Kip2^ acts as proliferation checkpoint during brain development. However, this checkpoint may be override by up-regulated Id2 leading to hyper-proliferation and development of neuroblastoma [155].

Also p53 plays an important role in maintaining normal neural progenitor cells (NPCs) proliferation, and it acts as repressor of *Id2* gene expression by binding a conserved site within its promoter. In contrast, mutated p53 in glioma is associated to elevated *Id2* expression and thus to increased proliferation and self-renewal of glioma stem-like cells [112].

Id2 and Id4 play a crucial role in regulating the glioblastoma multiforme (GBM) stem-like cells differentiation, thus reducing their cancer initiating potential. Indeed, upon induction of differentiation with histone deacetylase inhibitors, Id2 and Id4 are up-regulated and promote neuronal/astroglial differentiation at the expense of oligodendroglial differentiation by antagonizing the oligodendroglial lineage-associated transcription factors (Olig) 1 and 2 at the transcriptional level [156].

Because of the important role of the Id proteins in neural development [157], their deregulation may not only be implicated in tumor formation but also in neurodevelpmental disorders. For example, as Id2 and Id4 inhibit oligodendroglia differentiation [62], which is required for remyelination, they might have a role in multiple sclerosis that is characterized by axonal demyelination [158, 159]. Furthermore, the Id proteins might be involved in the Rett-syndrome (RTT): indeed, the *Id* gene transcriptional repressor MECP2 is mutated in RTT, which leads to overexpression of the Id proteins and altered neuronal maturation [160].

Traumatic brain injury leads to enhanced BMP2 and Id3 expression in the stem cells niche of the subventricular zone. Id3, in turn, inhibits the bHLH-mediated transcriptional repression of various astrocyte-specific genes. This leads to the differentiation of neural stem/precursor cells into astrocytes [161].

## Immune system-related processes

The class I bHLH proteins E12, E47 and HEB, and the Id proteins are major players in B-cell and T-cell development [162–166]. For example, the Id proteins are highly expressed in progenitor B-cells, whereas they decrease during differentiation to pre-B cells and mature B-cells, which inversely correlates with bHLH activity [165]. Additionally, Id2 enhances erythroid development by affecting the activity of the Ets-domain protein PU.1, a regulator of erythromyeloid development, and of the zinc-finger protein GATA-1: Id2 is able to interact with PU.1 and prevent the PU.1-GATA-1 interaction, thus initiating the myeloid versus erythroid program [167].

Recently, Id3 has been correlated to the TGFβ- and interleukin (IL4)-mediated signaling that controls the differentiation of CD4^+^ IL9 producing helper T (T_H_9) cells. Indeed, TGFβ1 and IL4 act as *Id3* transcriptional repressors, which results in E-protein- and GATA-3-mediated activation of the *IL9* gene transcription and even in enhanced anti-tumor response of the T cells in a melanoma mouse model [168].

The Id proteins are not only crucial for the proper development of the immune system, but they are also involved in immune response and may play an important role in regulating immunoglobulin gene expression. Indeed, the Id proteins have been found to inhibit immunoglobulin class switch recombination (CSR) to IgE in activated B-cells in response to TGFβ1 [101, 163, 169], which suggests a protective role of the Id proteins to prevent harmful immune reactions like allergic hypersensitivity. Moreover, Id2 is up-regulated in CD8^+^ T-cells as well as in memory CD8^+^ T-cells during infection, whereas Id2-deficient CD8^+^ T-cells show altered expression of genes influencing survival as well as impaired memory formation in response to infection [170]. Also, loss of Id2 in T-cells during influenza virus infection and in a model of acute graft-versus-host disease (GVHD) has been shown to increase IL10 levels [171]. This suggests that the Id proteins play a role in regulating survival of mature T-cells.

Then, TGFβ- or IL6-mediated up-regulation of Id1 in bone-marrow-derived myeloid cells has been shown to induce immunosuppression during tumor progression by inhibiting myeloid differentiation, while inducing pathologically activated immature cells, like myeloid-derived suppressor cells, and regulatory T-cells [172]. This shows that Id1 plays a role in tumor-induced immunosuppression.

## Mammary gland development and disorders

Mammary epithelial cells are characterized by decreased Id1-protein expression upon treatment with differentiation signals, whereas they start to proliferate upon constitutive Id1 expression [173]. Unlike Id1, Id2 has been found to be highly expressed in differentiated mammary epithelial cells [174]. Instead, Id2 deficiency leads to impaired proliferation and survival of mammary epithelial cells and to defects in alveologenesis in pregnant mice, resulting in lactation defect [175]. During mammary gland development Id4 expression suppresses p38MAPK activation, thus promoting cell proliferation and preventing apoptosis [176].

## Skin development and disorders

The Id proteins regulate keratinocyte proliferation and differentiation. Id1, Id2, and Id3 are expressed in proliferating human primary keratinocytes, but they are down-regulated upon induction of differentiation [177]. In contrast, the Id proteins are further expressed in squamous cell carcinoma 9 (SCC9) and HaCaT cells also upon treatment with differentiation stimuli, as well as in SCC sections with poor up to high differentiation [177]. However, in the case of Id3 it has been reported that this protein induces apoptosis of SCC cells through Elk-1-mediated caspase-3/-8 activation. A truncated variant of Id3 lacking the N-terminus is even a stronger inducer of apoptosis, probably because of the loss of the Cdk2 Ser-5 phosphorylation site, which abolishes Cdk2 regulation of the Id3 function [178]. This observation has led to the question, whether Id3 might play a tumor suppressor role in SCC.

Up-regulation of Id1 has been observed upon skin injury [179] and in psoriatic skin [180], which favors cell migration and proliferation. However, Id-protein expression is down-regulated during wound repair, which is induced by activin, a member of the TGFβ family involved in skin morphogenesis and wound healing [99].

## Bone formation

The Id proteins are important for the regulation of osteoblast differentiation of mesenchymal stem cells and bone-matrix formation, which is mediated by BMP2/6/9. Indeed, the Id proteins promote the proliferation of early osteoblast progenitor cells upon BMP9 stimulation, however, they are down-regulated during the terminal differentiation of committed osteoblasts [181].

In the case of osteoclast differentiation the Id proteins are down-regulated by the TNF-related activation-induced cytokine TRANCE that induces osteoclast formation from monocytes/macrophages via different transcription factors, including the bHLH Mi transcription factor (MiTF): the latter may bind the promoter of the osteoclast-associated receptor *OSCAR*, whereas it may be inhibited upon sequestration by the Id proteins [182, 183]. RANKL-mediated osteoclastogenesis may be inhibited by Egr2, which is a positive regulator of the *Id* genes, whereas Egr2 knockdown decreases Id2 expression, thus enhancing osteoclastogenesis [184].

## Myogenesis

In the course of myogenesis, myoblasts are differentiated into myotubes. This process is regulated by the highly coordinated interplay of the myogenic regulatory factors MyoD, Myf-5/-6, myogenin, and Mrf4 (class II bHLH proteins) and their binding partners, the class I E proteins and the class V Id proteins. The transcription of muscle-specific target genes is activated by heterodimers formed between the myogenic regulatory factors and the E proteins. Id1, Id2 and Id3 prevent skeletal muscle differentiation by sequestering the E proteins, thus blocking the activity of MyoD and other myogenic bHLH proteins [15, 185]. This inhibition is overcome by p204, a p200 protein family member that binds the Id proteins and also triggers a decrease in their level, presumably by shuttling them from the nucleus to the cytoplasm and thus accelerating their degradation [68, 69]. Indeed, it has been reported that in proliferating C2C12 myoblasts MyoD and Id1 are co-localized in the nucleus, while in differentiated myotubes MyoD is located in the nucleus and Id1 in the cytoplasm [186]. Furthermore, it has been observed, that Id2 reduces the myogenic markers MyoD and myogenin in myoblasts, however, ENH1 overexpression restores myogenic differentiation by binding Id2 [67].

## Angiogenesis

The formation of new blood vessels out of preexisting ones, so called angiogenesis, is an important process during development and wound healing. First evidence of a role of the Id proteins in angiogenesis has been provided by a study on *Id1*/*Id3* double knockout mice embryos that died due to hemorrhage in the forebrain and absence of blood vessels in the neuroectoderm. Interestingly, already a partial reduction of the Id levels in adult mice may reduce vascularization, growth and metastasis of tumor xenografts [149].

Thrombospondin-1 *(TSP-1)* has been identified as a target gene for Id1-mediated transcriptional repression [146]. TSP-1 is a glycoprotein known to be a potent inhibitor of in vivo neovascularization and tumorigenesis. In contrast, Id1 promotes these two events by inhibiting the transcription of the *TSP-1* gene via a yet unknown mechanism.

Another key player in angiogenic events is the vascular endothelial growth factor (VEGF): loss of Id1 function has been shown to inhibit basic fibroblast growth factor- and VEGF-induced angiogenesis [146, 149].

## Granulopoiesis

Granulopoeisis is the differentiation of primitive blood precursors into granulocytes primarily within the bone marrow. It has been shown that Id1 is up-regulated during early granulopoiesis and down-regulated during final maturation. In contrast, Id2 is up-regulated in terminally differentiated granulocytes. Constitutive expression of Id1 or Id2 in CD34(+) cells leads to different responses, indicating a different role of the two proteins in granulopoiesis: Id1 inhibits eosinophil development while enhancing neutrophil differentiation, whereas Id2 promotes final maturation of both eosinophils and neutrophils [187].

## Stem cells

Stem cells are mother cells that have the potential to differentiate into any specialized cell type in the body. They are able to self-renew or multiply; additionally, they play an important role in development and in the repair system of adult organisms. The Id proteins are expressed by embryonic and somatic stem cells, and initiate stemness by enhancing proliferation and inhibiting differentiation [188–191]. For example Id1 sustains the hematopoietic stem cell (HSC) self-renewal by inhibition of differentiation and keeping the undifferentiated state [188]. Moreover, in vitro Id1, Id2, and Id3 have been shown to increase the self-renewal and proliferation potential of cortical neural stem cells (NSCs), while inhibiting neuronal differentiation [188, 192]. Indeed, the Id proteins are critical for the adhesion of NSCs to their niche, as they negatively regulate the bHLH-mediated activation of *Rap1GAP*, thus preserving the GTPase activity of Rap1, a regulator of cell adhesion. In contrast, decreased Id expression leads to stemness loss of NSCs that undergo premature differentiation along the neuronal and oligodendroglial lineages at the expense of the astrocytic lineage [193].

The Id2 protein has been also shown to up-regulate the bHLH transcription factor Hes1 that is important to prevent premature neurogenesis of NSCs [194]. In contrast, Id2 negatively regulates NSCs self-renewal in the developing mouse cortex [195]. Pax7, a regulator of skeletal muscle stem cells, inhibits the premature differentiation of quiescent satellite cells by inducing Id2 and Id3 expression [196].

The Id proteins also occur in cancer stem cells, for example in glioma stem-like cells (GSCs), where Id3 induction through the EGFR/Akt/Smad5 pathway leads to acquirement of GSCs characteristics and angiogenesis [197]. Id1 has been proposed to regulate normal and malignant mammary basal stem cells through Wnt/β-catenin/TCF/c-Myc pathway activation. Accordingly, overexpressed Id1 may lead to oncogenic transformation of mammary stem cells, which promotes cancer stem cell activity in breast cancer cells [198].

## Id proteins and cancer

In contrast to normal cells, cancer cells present dysregulated cellular pathways resulting in uncontrolled cell division and spreading to adjacent tissue (invasion) as well as to distant sites (metastasis). As the Id proteins are involved in cellular pathways regulating proliferation and differentiation, it is not surprising to find them contributing to tumor-related processes [92, 141].

## Id proteins are involved in tumorigenesis and tumor progression

Although the Id proteins do not strictly meet the classical definition of oncogenes, as no tumor-associated mutations in the *Id* genes have been observed, with the exception of *Id2* in the colorectal cancer cell line HRT-18 [82] and of *Id3* in Burkitt lymphoma [199], the fact that Id-protein up-regulation not only is mediated by oncogenes like Myc, Ras, and (EWS)-Ets, but it also negatively affects tumor suppressor pathways (e. g. p53, pRb), emphasizes their importance in tumorigenic events. Elevated Id mRNA and protein levels have been found in many tumor types [86, 113–115, 117–119, 200–204], in which they have been often associated with poor prognosis.

The major role of the Id proteins in tumorigenesis is likely to be mediated by the inhibition of bHLH transcription factors and cell differentiation; however, an alternative mechanism might involve a disturbing effect of the Id proteins on the centrosomes. Centrosomes are the primary microtubule organizing centers (MTOC) in mitotic and post-mitotic cells. They are located adjacent to the nucleus and are regulators of cell-cycle progression. It has been shown that a fraction of Id1, but not of the other Id proteins, localizes to the centrosomes and mitotic spindle poles via binding to S5a, causing abnormal centrosome and centriole numbers [205–207]. These defects in the centrosome duplication presumably contribute to genomic instability and tumor formation, as they decrease the accuracy of mitotic replication. This property of the Id1 protein has been attributed to the presence of its N-terminal and HLH regions.

**Fig. 6:**
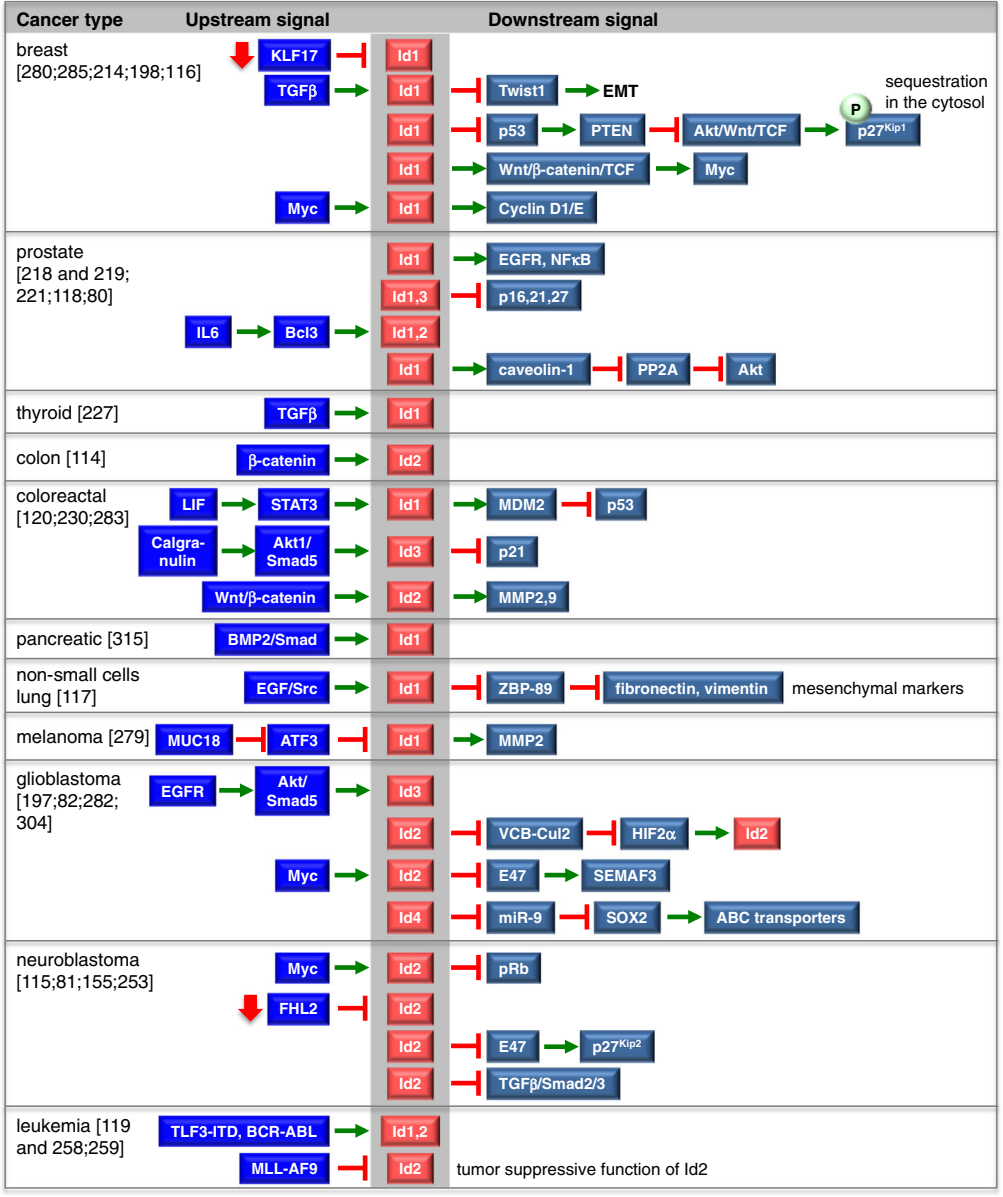
Upstream and downstream signals of the Id proteins in some cancer types

The role of the Id proteins in different types of cancer is briefly described in the following subsections and summarized in Fig. 6.

## Carcinoma

### Breast cancer

High levels of Id1 expression in several breast cancer cell lines are associated with high aggressiveness and invasiveness [116, 208–210]. Contrarily to Id1, Id2 is down-regulated in aggressive and invasive breast carcinomas, while it is up-regulated in differentiated breast cancer cells [211, 212]. Id1 expression may be induced by estrogen, which stimulates proliferation, whereas it may be down-regulated by progesterone. Accordingly, cells treated with Id1 antisense oligonucleotides proliferate slowly upon estrogen stimulation, whereas constitutive expression of Id1 abrogates the inhibition of breast-cancer cell growth by progesterone, which is known to block the growth and invasiveness of progestin-receptor positive cancers. These results indicate that Id1 overexpression can be, at least in part, responsible for the development of the hormone refractory stage of breast cancers [97].

Clinical studies have shown that breast cancer patients with negative estrogen receptor status are characterized by high Id1 expression, cell migration and poor prognosis [204]. In contrary, ERβ1 has been identified as a novel binding partner and inhibitor of Id1, which supports ERβ1-induced E-cadherin expression, resulting in the inhibition of the migration and invasion of breast cancer cells [213].

The tumorigenic role of Id1 in breast carcinogenesis has been associated to the inhibition of phosphatase and tensin homolog deleted on chromosome 10 (PTEN) at the transcriptional level through p53 down-regulation. Id3-mediated PTEN inhibition results in the activation of the Akt/Wnt/TCF pathway and in the cytosolic sequestration of p27^Kip1^ by its phosphorylation [214].

### Prostate cancer

It has been proposed that in androgen-dependent prostate cancers androgen might regulate proliferation, apoptosis and tumor suppression via Id1/Id3, Id2 and Id4 regulation, respectively. In contrast, lack of this regulation in androgen-independent cancers might lead to cell proliferation (Id1 and Id3 up-regulation), cell survival (Id2 down-regulation) and decreased tumor suppression (Id4 down-regulation) [215–217]. Additionally, overexpression of Id1 activates EGFR and NF-κB, resulting in aggressive tumor progression [218, 219]. Interestingly, it has been suggested that low levels of PSA and PSAP in aggressive prostate cancer might be due to Id1 overexpression [220]. Furthermore, up-regulated Id1 and Id3 expression decreases all three cell-cycle inhibitors p16, p21, and p27, leading to increased cell proliferation [221].

While Id4 is highly expressed in normal prostate epithelial cells, it decreases in prostate cancer cells in a stage-dependent manner and correlating with *Id4* promoter hypermethylation trough enhancer of Zeste 2 (EZH2)-dependent trimethylation of histone 3 at lysine 27 [222], which results in no Id4 expression in high-grade cancers. Accordingly, whereas knockdown of *Id4* supports the development of castration-resistant prostate cancer through constitutive activation of the androgen receptor [223], induced Id4 overexpression in highly malignant prostate cancer cells leads to apoptosis, decreased cell proliferation, migration and reduced tumor growth of subcutaneous xenografts [224]. These observations indicate that Id4 may act as a tumor suppressor, likely by binding Id1-3 with consequent reactivation of the E-protein-mediated DNA transcription [63] and/or by positive regulation of the expression and tumor-suppressor function of the androgen receptor [224]. However, it has been also reported that primary prostate cancers display high levels of Id4, which has been suggested to favor distant metastasis [225].

Caveolin-1, a cell membrane protein and positive regulator of cell survival and metastasis in prostate cancer, may interact with the helix-loop-helix domain of Id1. In prostate cancer cells this interaction plays an important role in epithelial-to-mesenchymal transition (EMT), it increases cell migration and taxol-induced apoptosis resistance through the activation of the Akt pathway [80]. Indeed, Id1 binding to caveolin-1 seems to improve the ability of the latter to bind and inhibit PP2A, which results in Akt activation [80].

### Cervical cancer

Because of the correlation of Id1 expression and human papillomavirus (HPV)-induced cervical cancer, it is suggested, that Id1 also plays a role in HPV-related cervical carcinogenesis [226].

### Thyroid cancer

In thyroid cancers the Id1 protein has been found to be overexpressed as an early target of TGFβ. This correlates with the development of a mesenchymal phenotype with increased invasion capacity [227].

### Nasopharyngeal cancer

Up-regulation of Id1, together with the p65 subunit of NF-kB, has been proposed to be a marker for poor prognosis in nasopharyngeal carcinoma. Accordingly, inhibition of Id1 and p65 with shRNA leads to down-regulation of MMP9 and reduction of tumor cell migration [228].

### Colon cancer

In colon cancers, which are mostly caused by mutations in the *APC* (adenomatous polyposis coli) gene and/or β-catenin genes, overexpression of Id2 has been observed and attributed to *Id2*-promoter activation by up-regulated β-catenin [114]. In primary colon cancer samples Id1 and Id3 regulate the self-renewal of colon cancer stem cells (CSCs) via p21. Id1/Id3 double knockout results in the lack of the tumor-initiating potential and increases the sensitivity of CSCs to the chemotherapeutic agent oxaliplatin [229].

### Colorectal cancer

Colorectal cancer cells overexpressing the leukemia inhibitory factor (LIF) are associated with chemotherapeutic resistance through down-regulation of p53. This is mediated by Stat3 activation, which in turn up-regulates Id1. The latter enhances MDM2 (mouse double minute 2 homolog), a key negative regulator of p53, leading to accelerated p53 degradation [120]. Also Id3 has been shown to promote colorectal tumorigenesis by inhibiting p21 [230]: indeed, Id3 is a downstream target of the Akt1/Smad5 pathway that is activated upstream by inflammation-induced up-regulation of the Ca^2+^ binding proteins calgranulin A and B (S100A8/9).

### Gastric cancer

Gastric adenocarcinoma shows Id1 up-regulation, whereas the metastatic tumors express lower Id1 levels than the primary tumors, suggesting that Id1 may not be determinant for gastric cancer metastasis [231].

### Hepatocellular cancer (HCC)

Id1 levels have been found to be high in HCC cells, whereas they are very low in normal liver tissues. Id1 overexpression, which is mediated by the MAPK/ERK pathway, is associated with increased c-Myc levels: indeed, Id1 knockdown leads to c-Myc reduction as well as c-Myc knockdown leads to Id1 reduction. Moreover, Id1 may interact directly with c-Myc without inhibiting the transcriptional activity of the latter [232].

Increased levels of Id1 have been also detected in tissue specimens from patients with cirrhosis without hepatocellular carcinoma and have been correlated with higher probability to develop the tumor [233].

### Pancreatic cancer

The Id proteins are implicated in the tumorigenesis of human pancreatic cancer, a highly malignant cancer type. Id2 is overexpressed in the cancer cells of the pancreatic tumor mass, contributing to cancer cell growth that can be inhibited by Id2 antisense oligonucleotides [234]. Also Id1 has been found to be overexpressed in human pancreatic cancers, which is associated with enhanced tumor angiogenesis but not with poor prognosis [235].

## Neural cancers

### Glioblastoma multiforme (GBM)

Glioblastoma multiforme (GBM) is the highest grade and most aggressive primary brain tumor. Id4 has been shown to inhibit glioma invasion in vitro by inhibiting MMP2 expression via an inhibitory interaction with Twist1 [236], a class II bHLH transcription factor that is highly expressed in GBM and is crucial for MMP2 expression [237, 238]. In addition, it has been shown that Id4 expression correlates with disease-free and overall survival of GBM patients [236]. In contrast, increased Id1-3 protein expression correlates with tumor vascularity, drug resistance and poor prognosis [239, 240]. Accordingly, *Id1*-silenced U87 GBM cells show significantly decreased proliferation and invasion capacity. Additionally, c-Myc, cyclin D1 and β-catenin expression decreases, while E-cadherin expression increases. As E-cadherin promotes mesenchymal-to-epithelial transition (MET), Id1 has been predicted to regulate the metastatic potential of GBM cells by supporting EMT [241].

High-grade glioma cells with high Id1 expression (but no Id2 and Id3 expression) show self-renewal capacity, whereas cells with low Id1 levels possess poor self-renewal capacity but proliferative potential. Undifferentiated low-Id1 cells are characterized by high expression of progenitor-associated markers like Olig2. Interestingly, both Id1-high and Id1-low cell types are able to generate high-grade gliomas in mice, with faster tumor development and higher penetrance in the case of the low-Id1 cells. Moreover, mouse survival significantly improves upon Olig2 but not Id1 deletion, suggesting that non-self-renewal glioma cells may have high impact on tumor growth [242]. In the case of mesenchymal gliomas, all three Id proteins, Id1-3, are required for maintaining high-grade gliomas, whereas deletion of these three genes leads to tumor regression through release of glioma initiating cells (GICs) from the perivascular tumor niche, which is mediated by the inhibition of the master regulator of cell adhesion Rap1. Therefore, the interaction of GICs with endothelial cells is disrupted, which results in their loss of self-renewal and tumor-forming capacity [243].

Recently, it has been shown that cancer stem cells and glioma aggressiveness are supported by a mechanism based on Id2 and the hypoxia-inducible factor 2α (HIF2α). Indeed, under hypoxia the unphosphorylated state of Id2 at Thr-27 is maintained upon inactivation of tyrosine-phosphorylation-regulated kinase 1 (Dyrk1). Unphosphorylated Id2 is able to bind the VHL-elongin-C complex, thus disrupting the ubiquitin-ligase complex VCB-Cul2 (pVHL-elongin C-elongin B-Cullin-2), which results in HIF2α stabilization by inhibition of its ubiquitination. This, in turn, leads to Id2 up-regulation, as HIF2α is a positive transcriptional regulator of the *Id2* gene [82].

### Neuroblastoma

In neuroblastoma, an extracranial cancer mostly occurring in infants and children and characterized by the amplification and overexpression of the oncogene *N-Myc* (neuronal *Myc*) [244], *Id2* is transcriptionally activated by Myc oncoproteins [115]. In turn, overexpression of Id2 abrogates pRb-induced cell-cycle arrest by direct binding to pRb [115, 141, 245]. However, whether a correlation exists between N-Myc and Id2 gene/protein expression in neuroblastoma [246–250], or if the Myc action in vivo is mediated by Id2 in other cancer types like epidermal neoplasia [251] and lymphomagenesis [252] are still controversial and would need further investigation [141].

The four-and-a-half LIM-only protein 2 (FHL2) is able to interact with all four Id proteins through a relatively conserved region within the N-terminal Id protein domains (C/S-L-S/A-E/D---S-L/V-A/G-I/G-S/A-R/A). After retinoic acid induced differentiation of neuroblastoma cells, FHL2 expression increases and counteracts the inhibitory effects of the Id proteins on E47, resulting in restored E47 induced transcription. Therefore, FHL2 is proposed to be a repressor of the oncogenic activity of Id2 in neuroblastoma [81].

Neuroblastoma cells are able to undergo reversible adaptive plasticity to survive and escape radio- or chemotherapy. One phenotype is highly proliferative and anchorage dependent (AD), the other is slow growing, anoikis-resistant and anchorage independent (AI) [253]. In the proliferating AD cells Id2 has been found to be 20-fold more expressed than in the AI cells (together with N-Myc, which would support Id2 being an effector of N-Myc [115, 245]), and it is suggested to support proliferation by antagonizing the TGFβ/Smad2/3 pathway. Accordingly, Id2 down-regulation in AD cells activates the TGFβ pathway, resulting, however, not only in decreased proliferation and induction of apoptosis, but also in activation of anoikis-resistant pathways, similar to cells with the AI phenotype. Instead, overexpressed Id2 in AI cells leads to the proliferative AD phenotype, allowing the cells to survive unfavorable and stressful conditions. These observations suggest that Id2 plays a key role in reversible adaptive plasticity in neuroblastoma cells. Simultaneous targeting of the AD and AI phenotypes by using the chemotherapy agents doxorubicin and metformin and of the pathways responsible for reversible adaptive plasticity with LY2109761, a TGFβ receptor inhibitor, and sorafenib, a multi-kinase inhibitor, results in decreased tumor growth and prolonged survival in established mouse neuroblastoma tumors [254].

### Medulloblastoma

In medulloblastoma the Id2 and Id3 proteins are overexpressed and promote tumor cell proliferation, whereas the Id1 protein has been found to be expressed in the tumor vessels, thus promoting tumor angiogenesis. In contrast, Id4 has been detected neither in normal cerebellum nor in tumor cells [255].

## Leukemia

### Acute myeloid leukemia (AML)

Id2 and Id3 show different expression patterns and subcellular localization in acute leukemia subtypes: for example, AML is characterized by higher Id2 and Id3 expression than acute lymphoblastic leukemia (ALL) [256].

Overexpressed Id1 or Id3 are able to immortalize growth factor-dependent hematopoietic progenitors resulting in cells with an acute myeloid leukemia (AML)-like morphology and decreased p15^INK^, p16^INK4^, p19^ARF^ and p21^Cip1^ in vitro. In vivo Id1 overexpression leads to lethal myeloproliferative disease [257]. *Id1* and *Id2* mRNA levels are associated with AML, whereby patients with increasing *Id1* levels correlate with poor clinical outcome. Microarray analysis suggests that *Id1* and *Id2* gene expression might be induced downstream of multiple signal transduction pathways of mutationally activated oncogenic tyrosine kinases like FLT3, TEL-ABL, BCR-ABL and PDGFRB [119, 258].

Down-regulated Id2 and up-regulated E2-2 have been observed in mixed lineage leukemia (MLL)-rearranged AML, which supports leukemia stem cell potential and confer poor prognosis. This suggests that Id2 might have a tumor suppressor role in MLL-rearranged AML as well as in t(8;21) AML [259].

*Id4* methylation plays an important role in disease progression in patients with myelodysplastic syndrome (MDS) that is a myeloid hematopoietic malignant disorder with high susceptibility to transform into AML. High levels of *Id4* methylation have been correlated with decreased survival [260].

### Chronic lymphocytic leukemia (CLL)

Id2 and Id3 support survival of CLL cells, probably by inhibiting pro-apoptotic pathways. Moreover, the CLL cells with high Id3 and, to a lesser extent, Id2 levels display high chemoresistance [261].

### Chronic myeloid leukemia (CML)

In CML *Id4*-promoter methylation increases during disease progression from the chronic to the accelerated phase and blast crisis [262]. The crucial role of Id4 has been proven with haploid loss of *Id4* in non-transformed TCL1-positive B cells, leading to enhanced B-cell proliferation and decrease in dexamethasone-mediated apoptosis [263].

## Lymphoma

Id2 is overexpressed in Hodgkin lymphoma tumor cells and suppresses the expression of B cell specific genes [264]. *Id4* methylation has been found to be high in lymphoma tissues, whereas no methylation has been detected in control tissues. Moreover, high *Id4* methylation correlates with decreased survival [265, 266]. In Burkitt lymphoma missense mutations of *Id3* have been found, which lead to mutated HLH domain and altered ability of Id3 to inhibit *TCF3* and/or *TCF4* [199]. Accordingly, *Id3* mutations have been observed in more than 50% of all Burkitt lymphomas, and the presence of *Id3* and/or *TCF3* mutations has been detected in 70% of sporadic Burkitt lymphomas. The Id3 destructive and/or the TCF3 activating mutations lead to TCF3 activated transcription resulting in pro-survival phosphoinositide 3-kinase (PI3K) signaling [267]. These results indicate that *Id3* inactivating mutations, together with IG-Myc translocation, are characteristic properties of Burkitt lymphoma pathogenesis [199].

## The role of the Id1 isoform in cancer

While there are many studies about the role of Id1 in cancer development and progression, much less is known about the Id1 isoform generated by alternative splicing. In contrast to the crucial role of Id1 in cancer, overexpression of its isoform in lung and prostate cancer cells leads to cell-growth arrest, tumor shrinkage, impaired angiogenesis and sensitization to radiotherapy-induced cell death [36]. Furthermore, unlike Id1 that promotes cell proliferation, its isoform causes a cancer stem cell-like phenotype and promotes its self-renewal. This would support a role of Id1 and its isoform in tumor initiation by promoting self-renewal properties by the spliced Id1 variant and, subsequently, proliferation by canonical Id1 [35].

## The role of Id4 in cancer

In various human tumor types such as acute and chronic leukemia [268], different malignant lymphomas [265, 266, 269], colorectal carcinoma [270], breast cancer [271] and gastric carcinoma [272], the tumor suppressor activity of Id4 is abrogated through epigenetic inactivation of its promoter by methylation during cancer development. These findings would suggest that *Id4* gene methylation degree might be used as a tumor marker. However, the role of Id4 in breast cancer remains controversial [273]: indeed, Id4 has been detected in breast cancer cells expressing p53 mutants, promoting tumor neo-angiogenesis [111], as well as in tamoxifen-refractory breast cancer, thus supporting chemoresistance [274].

## The role of the Id proteins in cancer metabolism

A crucial event of cancer development and progression is the metabolic reprogramming of cancer cells to cover their high glucose requirements. This takes place under control of oncogenic signaling pathways and several mutations occurring in cancer [275, 276]. For example, c-Myc seems to be one of the main regulators of aerobic glycolysis and glutaminolysis [277]. It has been shown that Id1 and c-Myc positively regulate each other’s expression in hepatocellular carcinoma cells and promote c-Myc-mediated glycolysis under aerobic conditions. Instead, under anaerobic conditions glycolysis is promoted by the hypoxia-inducible factor 1α (HIF1α) that recruits Mxi1, a transcriptional suppressor of Id1 and c-Myc, thus leading to down-regulation of Id1 and c-Myc expression [232].

## The role of the Id proteins in tumor angiogenesis and metastasis

Prerequisite for tumor progression and metastasis is a sufficient blood supply guaranteed by the formation of new blood vessels (tumor angiogenesis). The initiation of angiogenesis in tumors is triggered by the up-regulation of VEGF that promotes the exponential growth of the tumor. Like in neoangiogenesis during normal development, the Id proteins play a role also in tumor neoangiogenesis [149, 185]. Accordingly, *Id1+/− Id3−/−* mice fail to grow tumors due to poor vascularization and necrosis [149].

Matrix metalloproteinases (MMPs) are zinc-dependent endopeptidases that mediate membrane degradation and cell migration. Id1, Id2 and Id3 may increase MMP gene expression, leading to tumor cell invasion. High levels of Id1 and the membrane-type 1-MMP (MT1-MMP) [209] or MMP1 [278] have been associated to breast cancer metastasis. In melanoma, the Id1-induced up-regulation of MMP2 is mediated by the adhesion molecule MUC18. MUC18 positively regulates Id1 expression through the modulation of ATF3, contributing to melanoma metastasis. Indeed, silencing of MUC18 leads to increased ATF3 binding to the *Id1* promoter, which results in Id1 down-regulation [279].

The zinc-finger protein KLF17 is a metastasis suppressor by inhibition of *Id1* transcription upon binding to its promoter region. *KFL17* is significantly down-regulated in primary human breast cancer samples. Therefore, its suppression leads to Id1 induction, which might promote primary tumor vascularization via VEGF production, breast cancer cell invasion and EMT [280]. Id2 also seems to play a crucial role in tumor cell migration and invasion: indeed, c-Myc up-regulation and subsequent Id2 overexpression in highly metastatic human tumor cell lines lead to down-regulation of semaphorin 3 F (SEMA3F) that is a potent metastasis inhibitor and a direct target gene of the E47/Id2 pathway [281, 282].

In colorectal cancer it has been shown that hypoxia, a common feature of solid tumors, may increase the cancer stem cells (CSCs) subpopulations as well as promote cancer metastasis. The proposed mechanism relies on the hypoxia-mediated activation of the Wnt/β-catenin signaling that leads to Id2 overexpression that, in turn, induces a CSCs phenotype and expression of MMP2 and MMP9 responsible for increased cell migration [283].

There is growing evidence that phenotypic plasticity, in particular the epithelia-to-mesenchymal and mesenchymal-to-epithelial transition switch (EMT-MET), is required for effective cancer metastasis [284]. In breast cancer Id1 plays a crucial role in phenotype switching during lung metastasis [285, 286]. TGFβ-induced overexpression of Id1 is necessary not only to obtain tumor-initiating cells at the primary site, but also to switch the EMT phenotype, which is induced by the zinc finger transcription factor Snail at the primary site, back to the MET one at the colonization site. It has been shown that Id1 may induce the EMT-to-MET switch at the distant site by antagonizing Twist1, but not at the primary site, where the EMT phenotype is maintained by the presence of Snail [285].

## Id proteins and chemotherapeutic drug resistance

Chemotherapeutic drugs function by inducing cell death in cancer cells. A limitation of chemotherapy is the drug resistance that is associated with a more aggressive cancer disease and the resistance to further chemotherapeutic treatments. Factors positively affecting multi-drug resistance include up-regulation of the multi-drug transporter P-glycoprotein [287], and of the inhibitor of apoptosis Bcl2 [288], as well as activation of the Raf-1/MAPK [289] and NF-κB [290] pathways, and inactivation of the c-Jun N-terminal kinase (JNK) pathway [291]. Based on the fact that Id1 can activate the Raf-1/MAPK and NF-κB pathways [140, 292], a role of Id1 in the development of drug resistance has been suggested.

In prostate cancer Bcl3 is overexpressed via IL6, leading to the up-regulation of Id1 and Id2, and inducing resistance against anticancer drugs. Accordingly, Bcl3 knockdown results in decreased Id1 and Id2 expression, with tumor cells becoming more sensitive to chemotherapeutic drug-induced apoptosis [118]. REIC/Dickkopf-3 (Dkk-3) is a tumor suppressor that is reduced in numerous human cancers. Overexpression of REIC/Dkk-3 in malignant mesothelioma (MM) down-regulates Id1 expression via activation of ATF3 and Smad, resulting in enhanced JNK phosphorylation and REIC/Dkk-3-induced apoptosis [293]. In contrast, ectopic Id1 expression induces resistance to taxol treatment in breast, prostate and nasopharyngeal carcinoma cells [294–296]. Hence, increased sensitivity to taxol-mediated JNK activation and apoptosis in prostate cancer could be reached by using small RNA interfering technology to down-regulate Id1 [297].

Besides resistance against taxol, Id1 seems to induce resistance also against other antitumor agents including doxorubicin, cyclophosphamide [298] and epirubicin [299], which suggests that Id1 promotes cell survival by acting as universal antiapoptotic factor [300]. These results provide a linkage between up-regulation of the Id proteins and poor prognosis and severity of some human cancer types.

Id1 overexpression shows not only high correlation with tumor invasion, metastasis and poor prognosis in esophageal squamous cell carcinoma (ESCC), but it also plays a crucial role in the resistance to the anticancer drugs etoposide [301] and 5-fluorouracil (5-FU) [302]: indeed, etoposide enhances c-Jun/c-Fos expression that leads to *Id1* gene transcription and expression, resulting in inhibition of apoptosis [301]. 5-FU chemoresistance is accompanied by up-regulated expression of Id1, insulin-like growth factor 2 (IGF2) and the transcription factor E2F1. Id1 inhibits E2F1 degradation by binding to Cdc20. In turn, E2F1 binds to the *IGF2* promoter and activates its transcription. IGF2 increases phosphorylated-Akt and its downstream target thymidylate synthase, which abolishes 5-FU-induced apoptosis [302].

Treatment of hepatocellular carcinoma cells with the antitumor drug sodium butyrate (NaB), a histone deacetylase (HDAC) inhibitor, has shown dependency from the Id2 expression: indeed, NaB-mediated induction of anti-apoptotic Bcl2 is inhibited by Id2 knockdown but it is supported by Id2 overexpression. Therefore, the Id2 level has been suggested to serve as prognostic marker for clinical response to HDAC inhibitors [303].

Glioma stem cells (GSCs) are relatively resistant to chemotherapy and irradiation. Id4 has been shown to suppress miR-9 and induce SOX2. Enhanced SOX2 expression leads to induction of ATP-binding cassette (ABC) transporters 3 and 6, resulting in chemoresistance of GSCs. Furthermore, elevated SOX2 expression dedifferentiates astrocytes and glioma cells to GSCs [304].

## The Id proteins as therapeutic targets

Cancer diseases are one of the most frequent causes of death in developed countries and require the constant research of novel, potent anti-tumor therapeutics. The Id proteins represent interesting targets for such purpose, as they are involved in cellular key events related both to tumorigenesis and cancer progression [149, 201, 305].

Different approaches to reduce aberrant Id-protein levels and restore differentiation of hyperproliferative cells have been successfully applied. For example, by treating human metastatic breast cancer cells with an Id1 antisense oligonucleotide not only Id1 decreases, but also MT1-MMP. Therefore, the significantly reduced breast cancer metastasis to the lung might be a result of reduced MT1-MMP-mediated invasiveness [209].

Furthermore, targeting Id1 with Id1-siRNA in adenoid cystic carcinoma (ACCM) mouse models inhibits tumor growth, reduces tumor cell proliferation/invasion and induces apoptosis [306].

An *Id1* antisense oligonucleotide conjugated to a peptide that addresses tumor neovessels specifically has been used to decrease the growth rate of breast tumors and the highly aggressive Lewis Lung carcinomas (LLCs). Moreover, the metastatic growth of LLCs could be delayed. This antitumor effect enhances upon combination with the Hsp90 inhibitor 17-AAG [307].

MicroRNAs (miRNAs) are a class of small, non-coding RNAs that regulate gene expression and differentiation by interacting with mRNAs. Retinoic acid-induced up-regulation of two miRNAs (miR-9 and miR-103) during neuroblastoma cell differentiation inhibits Id2 expression and cell growth. Therefore, these two miRNAs may have tumor suppressive properties in several neural tumors [308].

In metastatic breast cancer, glioblastoma and salivary gland cancer cannabidiol, a low toxic cannanbinoid, has been shown to reduce Id1 expression, resulting in less tumor growth, aggressiveness and metastasis [309–311].

The influence of MK615, an extract from the Japanese apricot “*Prunus mume*” known for antitumorigenic and antiinflammatory effects, has been studied in human malignant melanoma cells: MK615 reduces Id1 expression and, therefore, cell growth through the inhibition of the ERK1/2 pathway [312].

Curcumin significantly down-regulates mRNA and protein levels of Id1 in prostate cancer cells and xenografted tumors, which is accompanied by induction of apoptosis and tumor growth suppression [313].

Berberine, an isoquinoline alkaloid present in different herbs, including barberry, has shown anti-proliferative and anti-metastatic effects in hepatocellular carcinoma mice via Id1 down-regulation at the transcriptional level. Indeed, berberine may inhibits the *Id1* promoter activity, resulting in suppression of cellular growth, invasiveness and VEGF secretion [314].

An arabinogalactan polysaccharide from the *Panax notoginseng* (RN1) has been able to inhibit microvessel formation in pancreatic cancer-cell xenograft tumors in nude mice through the inhibition of BMP2/Smad-induced Id1 expression [315].

2-Methoxyestradiol, a metabolite of 17-β-estradiol, may inhibit angiogenesis and reduce tumor growth at late stages through enhanced apoptosis. This correlates with the inhibition of Id1 in mouse and human breast cancer cell lines [316]. In gastric cancer cells, Id1 could be dose-dependently decreased by sulindac sulfide, a non-steroidal anti-inflammatory drug [317]. Furthermore, treatment with epigallocatechin-3-gallate, a catechin from tea, down-regulates Id1 mRNA and protein in poorly differentiated AGS gastric cancer cells [318].

TGFβ, Id1 and CD44 regulate glioma stem cells, which are responsible for glioblastoma initiation, relapse, and therapeutic resistance. Treating patient-derived glioblastoma specimens with LY2109761, a TGFβ receptor type I and II dual antagonist, leads to the reduction of CD44^high^/Id1^high^ glioma stem cells, thus preventing tumor initiation and recurrence [109].

USP1 is up-regulated by PDGF via E2F. This signal stabilizes Id2 expression that is crucial for glioma survival. Pimozide, an anti-psychotic drug and USP1 inhibitor, might have therapeutic activity in patients with proneural PDGF-driven glioblastoma. Indeed, pimozide suppresses Id2 expression and reduces tumor growth [319].

Furthermore, chemosensitivity of human U87 GBM cells may be enhanced by silencing *Id2*. Indeed, the antiproliferative effect of the anti-tumor agents semustine, teniposide and temozolomide is higher in *Id2*-silenced cells than in Id2-expressing cells. As *Id2*-silenced cells express higher levels of caspase-3, it is likely that their increased chemosensitivity is due to the up-regulation of pro-apoptotic pathways [240].

Src tyrosine kinase is suggested to promote tumor aggressiveness through BMP2-induced Id1 expression. Accordingly, Id1 levels are reduced in breast, prostate, lung and colon cancer cell lines treated with the small-molecule Src inhibitor AZD0530 [320].

PI3K/Akt signaling is a downstream component of Id1 and promotes osteosarcoma progression. Accordingly, the PI3K inhibitor LY294002 decreases Id1-induced osteosarcoma tumor growth [321].

Inhibition of BMP signaling by the small molecule DMH2 that binds BMP type I receptors has led to decrease in Id proteins and suppression of growth of cancer cells expressing stem cell markers [322].

The DNA-damaging agents camptothecin and adriamycin are able to inhibit Id1 expression trough wild-type p53 induced DEC1 that binds to the* Id1* promoter and represses its transcription [138]. 

Recently, a small molecule (AGX51) that directly targets the Id proteins has been discovered, which blocks tumor angiogenesis and is currently in the preclinical phase for drug development [323, 324]. In addition, peptide-based molecules have been developed to bind the Id proteins and inhibit their function. A peptide aptamer (Id1/Id3-PA7) has been developed, which induces cell cycle arrest and apoptosis in ovarian and breast cancer cells by inhibition of Id1 and Id3 [325, 326]. A modified HEB HLH domain has been shown to interfere with and inhibit Id2 in human neuroblastoma cells. This has led to the activation of the cell-cycle inhibitor p27^Kip1^ with induction of growth arrest and neural differentiation [327, 328]. Short peptides based on MyoD [329] or Id [57, 330] HLH sequences have been designed, which bind the Id proteins in the low-micromolar range and are able to inhibit proliferation of cancer cells and of a vascular smooth muscle cell phenotype.

A T-cell mediated vaccine approach has been recently tested in mouse neuroblastoma. *Id2*-knockdown neuroblastoma cells (Id2kd-Neuro2a) have shown to be immunogenic. These cells do not grow in immune-competent mice that even develop immunity against wild-type neuroblastoma cells. In contrast, Id2kd-Neuro2a cells grow aggressively in immune-compromised mice. In combination with the use of an antibody against the cytotoxic T lymphocyte antigen-4 (CTLA-4), an inhibitor of T-cell responses, vaccination with Id2kd-Neuro2a cells of mice bearing established neuroblastoma tumors has been shown not only to suppress the tumor growth but also to eradicate the tumor itself [331].

A nanoparticle-based approach has been recently used to deliver recombinant Id4 protein as a biotherapeutic agent into prostate cancer cells or into prostate cancers in mice. Indeed, *Id4* expression is epigenetically silenced in prostate cancer, whereas its ectopic expression suppresses the cancer phenotype. Accordingly, delivery of Id4 encapsulated in biodegradable polycaprolactone/maltodextrin nanoparticles leads to increased apoptosis, decreased proliferation and colony formation. In vivo the Id4-nanoparticle approach has been shown to be more efficient than the administration of docetaxel in reducing the tumor volume [332].

## Conclusions

The Id proteins exert their function by interacting with and modulating key regulators of the cell cycle. The four members of the Id family show distinct expression patterns and, despite the high homology of their HLH domains, display different protein-protein interaction preferences both under physiological and pathological conditions. This suggests that, on the one hand, the structure of the Id HLH domain is fine-tuned and that, on the other hand, the non-conserved N-terminal and C-terminal domains are determinant for the specific protein binding profile of each Id protein. Although the main mechanism of the Id proteins rely on the direct interaction and modulation of bHLH factors, the data reported in the literature and partly summarized in this review clearly show that the mode of action of these four small proteins is highly complex. This is due to their ability to interact with a multiplicity of proteins that belong to different families and affect different cellular pathways. Strikingly, such functional diversity reflects the structural flexibility of the Id proteins that, in fact, contain high degree of disorder. Obviously, this significant structural and functional diversity makes the Id proteins a challenging topic of research; however, the successful work done so far encourages to continue the exploration of the biology and structure of these intriguing protein regulators, with the aim to understand and solve controversial data, to answer still open questions, to further validate them as targets for tumor diagnostics and therapy, and to develop drug-like molecules for their detection and inhibition in vivo.

## Abbreviations

AML: Acute myeloid leukemia
Bcl: B-cell leukemia
bHLH: basic-HLH
CRMP1: Chromosome region maintenance protein 1
CSC: Cancer stem cell
CSN: COP9 signalosome
EMT: Epithelial-to-mesenchymal transition
ENH: Enigma homolog protein
EZH2: Enhancer of Zeste 2
FHL2: Four-and-a-half LIM-only protein 2
GBM: Glioblastoma multiforme
GSC: Glioblastoma-derived stem cell
H3K9ac: Acetylation of histone H3 at lysine 9
H3K9me2: Dimethylation of histone H3 at lysine 9
HBX: Hepatitis B virus-encoded protein X
HIF: Hypoxia-inducible factor
Id: Inhibitor of DNA binding and cell differentiation
IG: Immunoglobulin
LIF: Leukemia inhibitory factor
MAPK: Mitogen-activated protein kinase
MDM2: Mouse double minute 2 homolog
MET: Mesenchymal-to-epithelial transition
MLL: Mixed lineage leukemia
MMP: Matrix metalloproteinase
MT1-MMP: Membrane-type 1 MMP
NES: Nuclear export signal
NF-Y: Nuclear factor Y
NLS: Nuclear localization signal
NMR: Nuclear magnetic resonance
PAS: Per-ARNT-SIM
PDGF: Platelet-derived growth factor
PSA: Kallikrein3/prostate specific antigen
PSAP: Prostate acid phosphatase
PTEN: Phosphatase and tensin homolog deleted on chromosome 10
S100A8: Ca^2^^+^ binding protein calgranulin A
SCC: Squamous cell carcinoma
TCF: T-cell factor
TCR: T-cell receptor
TNF: Tumor necrosis factor
